# Virus Neutralisation: New Insights from Kinetic Neutralisation Curves

**DOI:** 10.1371/journal.pcbi.1002900

**Published:** 2013-02-28

**Authors:** Carsten Magnus

**Affiliations:** Institute for Emerging Infections, Department of Zoology, University of Oxford, Oxford, United Kingdom; University of Texas at Austin, United States of America

## Abstract

Antibodies binding to the surface of virions can lead to virus neutralisation. Different theories have been proposed to determine the number of antibodies that must bind to a virion for neutralisation. Early models are based on chemical binding kinetics. Applying these models lead to very low estimates of the number of antibodies needed for neutralisation. In contrast, according to the more conceptual approach of stoichiometries in virology a much higher number of antibodies is required for virus neutralisation by antibodies. Here, we combine chemical binding kinetics with (virological) stoichiometries to better explain virus neutralisation by antibody binding. This framework is in agreement with published data on the neutralisation of the human immunodeficiency virus. Knowing antibody reaction constants, our model allows us to estimate stoichiometrical parameters from kinetic neutralisation curves. In addition, we can identify important parameters that will make further analysis of kinetic neutralisation curves more valuable in the context of estimating stoichiometries. Our model gives a more subtle explanation of kinetic neutralisation curves in terms of single-hit and multi-hit kinetics.

## Author Summary

How many antibodies have to bind to a virus particle such that it is prevented from infecting a cell? This seemingly simple question has not been answered yet. However, this number is crucial to determine whether a vaccine can stimulate the immune system to elicit enough antibodies to neutralise virus before starting an infection. Two different approaches have been applied to answer this question, leading to contradictory results. One approach is inspired by concepts from binding kinetics, the other approach is a more conceptual one. Here, I describe the advantages and disadvantages of either approaches and condense the advantages of both into one model framework. I show under which conditions the framework can be used to identify the number of neutralising antibodies. In addition, this model can explain why viruses might not completely loose their infection potential even when there is a huge excess of antibodies.

## Introduction

Antibodies are the most efficient way the immune system fights viruses before they infect host cells. Most of the available vaccines against viral pathogens stimulate the immune system to produce antibodies against a variety of molecular patterns on the viral surface, the *epitopes*. Each antibody response consists of many different antibodies directed against different epitopes or directed against the same epitope but varying in their binding strengths. During the first three quarters of the last century, these antibody mixtures were tested for their neutralising potential. However, rational vaccine design requires knowledge of which specific antibodies have the highest neutralising potential. A vaccine should then stimulate the immune system to produce these antibodies.

To study antibody binding and to characterise antibodies, different methods have been proposed. In the early days of virology, the number of antibodies required to neutralise a virion was studied for many different viruses, using the concepts of chemical binding kinetics. Antibodies were added to virion populations and at intervals of one or more minutes, samples were taken and immediately diluted. This stopped antibody binding and the surviving virions were counted in plaque assays [1]. The theory employed for interpreting the kinetic neutralisation curves is based on early work on western equine encephalitis virus and poliomyelitis virus [2]. The basic assumption in these models is that there is at least one critical binding site on the virion surface. The virion is neutralised as soon as one of these binding sites is bound to an antibody. The shape of the time-neutralisation curves was thought to carry information on the number of antibodies needed for virion neutralisation. A sudden decline in the time-neutralisation curve (no lag-phase), was interpreted as a single-hit mechanism, i.e. that the binding of one antibody is sufficient to neutralise the virion [2]. In contrast, a lag phase at the start of the time-neutralisation curve was interprereted as a multi-hit mechanism. Experimentally observed time-neutralisation curves decline to a certain level, further neutralisation does not seem possible. In the early framework, this leveling-out was interpreted as a persistent virus fraction that cannot be neutralised. According to this framework, the experimentally obtained plots were often interpreted as a proof for a single-hit mechanism [2]. By varying the experimental conditions, however, some evidence for multi-hit mechanisms arose (reviewed for example in [3], [4]).

McLain and Dimmock [5] used these methods to study the neutralisation of HIV and suggested that three antibodies can neutralise a single HIV-virion. Klasse and Sattentau [6] reviewed these low numbers critically and introduced the differentiation between binding kinetics and occupancy, i.e. the number of antibodies attached to a virion. They show that the minimum occupancy required for viral neutralisation only influences the slope of the binding kinetic curves and not necessarily the shape. Other evidence against a single-hit mechanism of neutralisation comes from imaging HIV-virions while they infect a cell [7]. It seems as if more than one HIV-spike interact with target cell receptors. An HIV-spike consists of three heterodimers (envelope protein, Env), each comprising one gp120 that is loosely attached to one surface embedded gp41 [8]–[10]. The spikes are also referred to as *trimers*. These trimers establish contact with target cell receptors and mediate infection of the cell [11]–[13]. This makes the trimers the perfect targets for neutralising antibodies [14], [15]. However, the contact region between the target cell and the virion seems to be relatively small in comparison to the virion's surface. One antibody could easily bind to a trimer that is not engaged in the contact region and this binding would not prevent the attachment process. Therefore, the concept of stoichiometries was introduced into virology. The question of how many antibodies must bind to a whole virion for neutralisation was broken down into studying the number of interactions of spikes and cellular receptors required for viral entry (*stoichiometry of entry*) and how many antibodies must bind to a single spike such that it loses functionality (*stoichiometry of (trimer) neutralisation*) [16]–[21]. One can then calculate the number of antibodies that have to bind to a single virion for neutralisation, including random binding effects [22]. Note that the term stoichiometry is not as strictly used in virology as in chemistry. In chemistry the term describes the quantitative relationship between reactants and products. By contrast, in virology, the term stoichiometry describes how many molecules are involved in certain processes.

The interpretation of neutralisation kinetic curves as single-hit neutralisation and the concepts of stoichiometry, in which many more antibodies have to bind for neutralisation, seem to contradict each other. Binding kinetics describe the change of compounds during a chemical reaction. This concept was transferred to describe antibody binding mechanisms for neutralisation kinetics. For chemical binding curves the concentration of the reactants are measured over time. In neutralisation kinetics one does not measure the concentration of antibody-virion complexes but the percentage of neutralised virions. This means that a second reaction is needed to predict neutralisation kinetics. Direct observation of the products is therefore not possible, making it error-prone to conclude single-hit kinetics out of neutralisation kinetics curves. The concept of occupancy and stoichiometry assumes that there is a minimal number of viral spikes that have to engage with cellular receptors to mediate entry. Further it is assumed that if antibodies knock out a sufficiently high number of spikes the virion is unable to infect any cell. This framework does not incorporate the antibody binding process and only informs about the numbers of antibodies that have to bind to a virion population for neutralisation. It cannot inform about the antibody concentrations that are needed to reduce the infectivity of a virion population.

Here we derive a mathematical framework combining the concepts of chemical binding kinetics and stoichiometries to study neutralisation kinetics. This framework makes it possible to predict neutralisation curves as obtained in earlier studies. Important parameters have not been measured so far, but we show how these parameters influence the predicted neutralisation curves.

## Models

In our model we combine antibody binding dynamics and stoichiometric requirements for neutralisation in two separate steps. The first step describes the binding of antibodies to spikes. In the second step we focus on the virions and check whether they are neutralised or not.

### Step I: Binding of antibodies to spikes

Whilst the formulation in the model section is generic in the sense that it is applicable to all enveloped viruses, the model is mainly inspired by the neutralisation of HIV by monoclonal IgG antibodies. In this system, it is unlikely (i) that an IgG antibody binds to two epitopes of the same spike, due to geometrical reasons, and (ii) that an antibody binds to two epitopes of neighbouring spikes, due to the low spike density [23], [24]. We therefore assume that one antibody binds with one of its two Fab-regions. Hence, we can describe binding of one antibody, 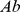, to one epitope of a spike, 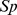, as a chemical reaction
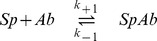
in which 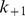 is the antibody binding constant and 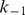 its dissociation constant of the first antibody binding. A spike with 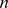 epitopes specific for one antibody type can bind to 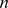 antibodies according to the chemical binding reaction
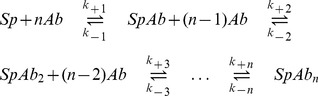
(1)with the binding constant 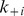 and dissociation constant 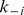 for forming and dissociation of the 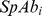 complex, respectively.

Employing the concepts of chemical kinetics, this reaction equation can be translated into the following set of differential equations [25]. Each equation describes the time evolution of the concentration of one of the components over time. Note that concentrations are indicated by square brackets.
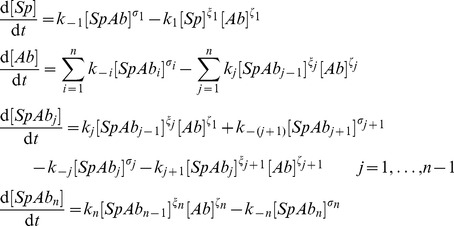
(2)


In these equations, the exponent 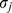 is the order of the dissociation reaction of the 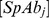 complex, the exponent 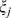 is the order of the 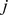
th reaction step with respect to the 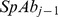 complex and the exponent 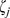 is the order of the 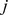
th reaction step with respect to the antibody, for 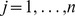. Note that we use the notation 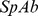 for 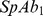 and 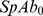 for 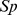, respectively.

### Step II: Neutralisation on the virion level

Each virion expresses a certain number of spikes on its surface. Equation 2 describes the binding to spikes as if they were in solution. However, the spikes are attached to viral surfaces. Zhu et al. [26] visualised 40 HIV-1 virions with cryo-electron microscopy and found that the number of spikes per surfaces varies from virion to virion with a mean expression of 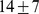 spikes per virion. Variation in spike numbers in other viruses might also occur. We therefore define the spike number distribution 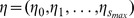 with 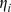 as the fraction of virions with 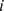 spikes, where 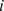 ranges from 0 spikes to the maximal spike number 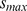. For each time step 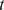 we calculate the concentration of spikes bound to 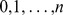 antibodies according to equation 2.

In the second step, the spikes are re-distributed to the virions. The fraction of spikes bound to 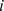 antibodies at time 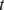, 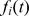 arises from the concentrations of spikes bound to 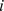 antibodies:
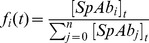
(3)for 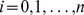. The concentrations are determined by (numerically) solving the system of ODEs in equation 2. For the sake of simplicity, we write 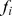 instead of 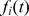 wherever possible. For given fractions of spikes bound to 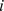 antibodies, 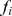, the probability that a virion with 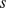 spikes has exactly 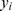 spikes bound to 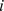 antibodies for 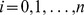 follows a multinomial distribution and is
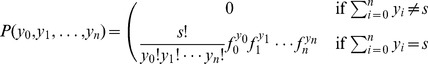
(4)A virion with fewer than 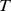 spikes is never infectious according to the definition of the stoichiometry of entry. If the spike number 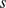 is at least 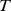, the virion is infectious if it has 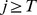 spikes with fewer than 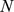 bound antibodies. Therefore, the total number of spikes with 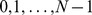 bound antibodies must sum up to 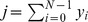 with 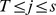 for the virion to be infectious.

**Figure 1:**
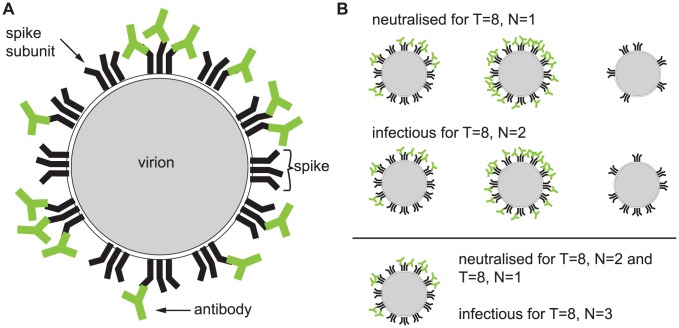
Illustration of the concept of stoichiometries and the parameters used in the model. The sketch in panel (A) depicts a virion with 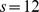 spikes each consisting of three identical subunits. Thus, each spike has 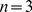 binding regions for one type of monoclonal antibodies. The virion has 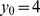 spikes bound to 0 antibodies, 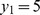 spikes bound to 1 antibody, 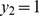 and 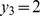 spikes bound to 2 and 3 antibodies, respectively. Under the assumptions that the stoichiometry of entry is 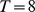 and the stoichiometry of neutralisation is 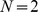, the virion is still infectious because it has nine spikes with fewer than two antibodies bound. Panel (B) shows several virions that are neutralised or infectious according to the definition of stoichiometries.

As an example, let us consider the virion sketched in Figure 1(A). It has 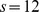 spikes each consisting of 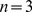 subunits (which again is inspired by the structure of an HIV virion). This virion has 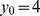 spikes bound to 0 antibodies, 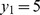 spikes bound to 1 antibody, 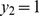 and 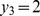 spikes bound to 2 and 3 antibodies, respectively. Let us assume that at time point 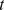 the concentration of spikes bound to one antibody, 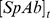, equals 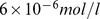, and the concentrations 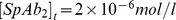, 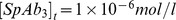 and the concentration of unbound spikes is 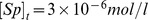. The fraction of spikes bound to 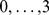 antibodies is then 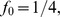 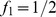 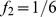 and 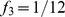. The probability that a virion with 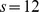 spikes has 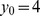 unbound spikes, and five, one, three spikes bound to one, two, three antibodies, respectively, given these concentration is 0.0117 = 1.17%.

To calculate the probability that a virion with 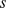 spikes is infectious we have to calculate the probability that a virion with 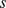 spikes has 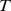 or more spikes with fewer than 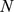 antibodies. Thus the probability that a virion with 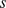 spikes is infectious is
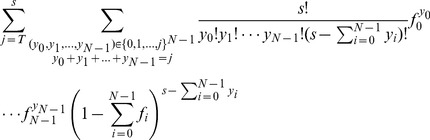
(5)


In the second sum we sum over all possibilities that the number of spikes with fewer than 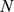 bound antibodies equals 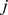. This sum can be re-written by going through all combinations of these numbers and reads then 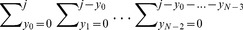. The *percent infectivity* of a viral stock at time 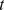, 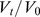 is the number of infectious virions at time 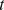, 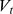, divided by the number of infectious virions at time 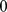, or without any bound antibody, 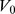. This quantity can be experimentally measured by plaques assays [2], [3] or in infectivity assays with pseudotyped virions [16]. To calculate 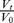, we weigh this probability (equation 5) with the probability that a virion has 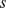 spikes, 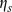. In addition, we have to divide by the probability that a virion has at least 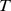 spikes, because the infectivity of a viral stock obtained with infectivity assays is always normalised with the infectivity of a viral stock without any antibodies. Thus we obtain:
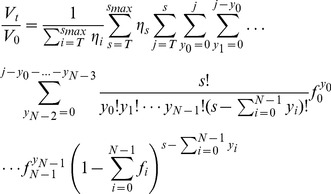
(6)where 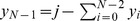 and 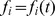 as defined in equation 3.

A remark about the units of the reaction constants: As concentration is measured in 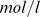, the product of reaction constants and product concentrations must have the unit 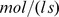 for every summand on the right hand side of the equations in Equation 2. The reaction kinetic equations are generic in the sense that they allow for any possible reaction order in any step with respect to any product. Thus the units of the reaction constants are 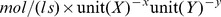 where 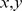 are the reaction orders in respect to the product 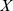 and 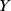, respectively. For simplicity, we omit the units in the following.

A summary of the parameters used in the models can be found in Table 1. All calculations are implemented in the R language for statistical computing [27] and are available in Dataset S1.

**Table 1:** Parameter definitions.

Parameter	Meaning
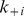	binding constant of the 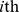 step
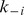	dissociation constant of the 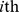 step
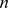	number of epitopes per spike specific for one monoclonal antibody
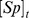	spike concentration at time 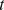 , the index 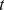 is omitted wherever possible
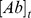	antibody concentration at time 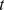 , the index 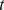 is omitted wherever possible
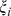	order of the 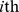 reaction step with respect to the 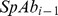 complex
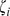	order of 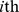 reaction step with respect to the antibody
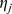	fraction of virions with 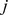 spikes
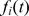	fraction of spikes bound to 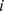 antibodies, 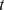 is omitted wherever possible
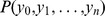	probability that a virion with 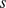 spikes has exactly 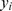 spikes bound to 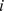 Abs
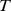	number of spike-receptor interactions needed for entry
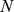	number of antibodies-epitope interactions required to neutralise one spike
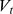	number of infective virions at time 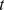
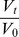	percent infectivity of a viral stock at time 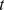

Definition of the parameters used in the model.

## Results

The input parameters of our model for the percent infectivity, 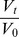, in equation 6 are the starting concentrations of spikes and antibodies, 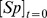 and 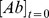, respectively, the number of binding sites for a particular antibody per spike, 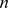, the spike number distribution 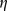, the stoichiometry of entry, 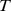, the stoichiometry of neutralisation 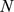, the association and dissociation constants 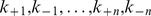 and the reaction orders 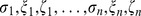. In the first subsection we reduce the number of variables by looking at the human immunodeficiency virus (HIV). In the second subsection we describe how the HIV-specific framework can be extended to study other viruses.

### HIV-specific model

HIV virions express trimers of the heterodimeric envelope proteins (Envs) gp120 and gp41 embedded on their surface [8]–[10]. As a monoclonal antibody binds to a well-defined region only present once per envelope subunit [14], up to three antibodies can bind to a whole spike, thus 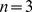. According to [24] the average distance between two spikes is bigger than the distance between two Fab-regions of one antibody, which lies in the range of 15 nm. In addition, the average distance between two epitopes must be smaller than the diameter of a trimer, which is 10.5 nm. Therefore intra-spike and inter-spike binding of two Fab regions of the same antibody is unlikely in the case of HIV. Hence we assume that each antibody binds to one epitope.

The additional binding of an antibody to a trimer that is already bound to one or two antibodies might be hindered, e.g. by sterical hindrance. This is reflected in the model by differences in the binding and dissociation constants.

Zhu et al. [26] counted the spike numbers of 40 HIV-1 virions and found a mean of 14 spikes with a variance of 49. However, the sample size is too small to take the fraction of virions with 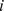 spikes as a measure for the real fractions. In an earlier publication [20], we therefore defined a discretised Beta-distribution with mean 14 and variance 49 and we will use this distribution for the HIV-specific model. This distribution was chosen because the Beta-distribution is defined on a closed set and has a high flexibility depending on two parameters which can be expressed in terms of mean and variance. The form of the distribution ranges from peaks at the edges to one peak in the centre. As we do not have an experimentally determined trimer distribution but only mean and variance, a discretised version of the beta distribution might come as close to the real distribution as possible.

### The effect of input parameters on the kinetic neutralisation curves

#### Reaction order

**Figure 2:**
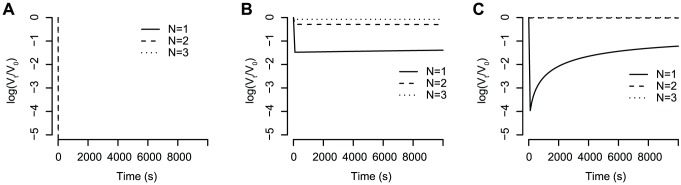
Predictions for kinetic neutralisation curves for the elementary reaction model. (A) All binding constants are 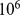 and all dissociation constants are 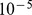. The stoichiometry of entry is assumed to be 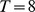. The starting concentration of antibodies is 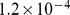 and the starting concentration of trimers is 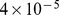. (B) Same constants as in (A) but the starting concentration of antibodies is 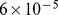. (C) The binding constants are 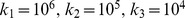 and the dissociation constants are all 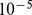. The stoichiometry of entry is 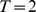 and the antibody starting concentration is 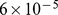.

**Figure 3:**
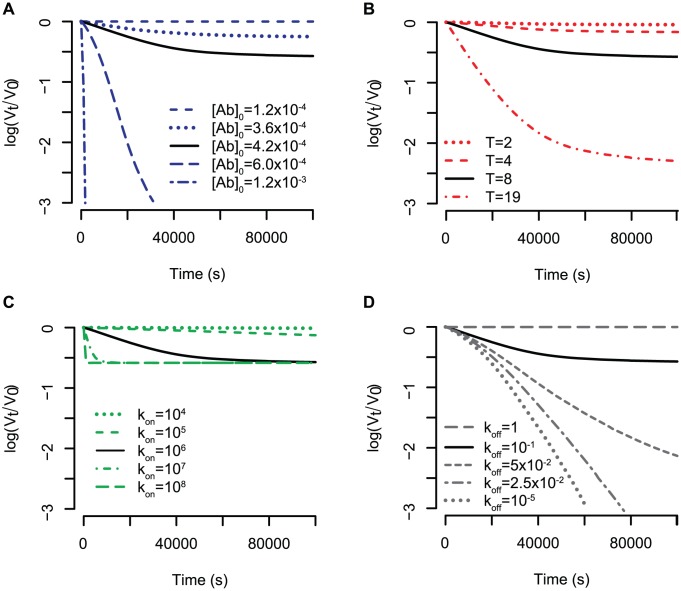
Influence of different parameters on the kinetic neutralisation curves. (A) Antibody starting concentration. The starting concentration of spikes is constant for all graphs, 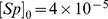. The stoichiometry of entry is 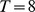 and the stoichiometry of trimer neutralisation 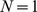. The binding constants are all 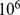 and the dissociation constants are all 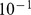. (B) Stoichiometry of entry. The parameters are the same as for (A) but the antibody starting concentration is 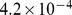. (C) and (D) Influence of the ratio between binding and dissociation constant in case all binding constants have the same value 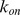 and all dissociation constants have the same value 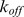. In (C) the ratio between the binding and dissociation rates is kept constant at 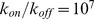 whereas in (D) the binding constant is kept constant at 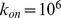.

**Figure 4:**
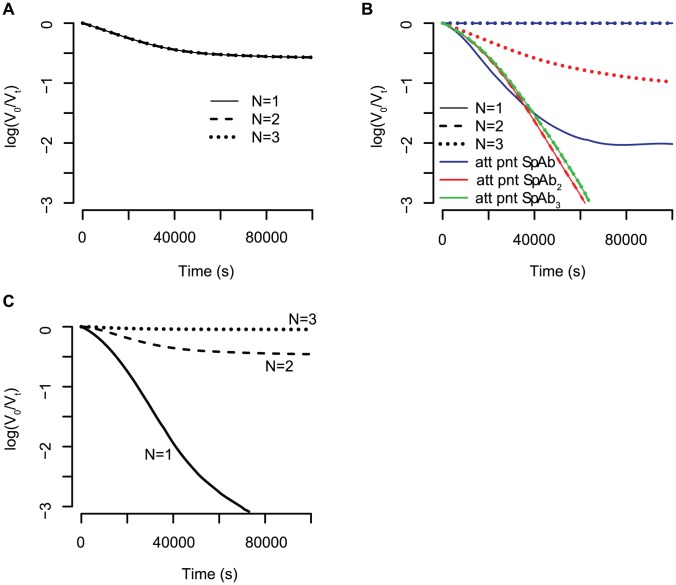
Influence of reaction parameters on the feasibility of estimating the stoichiometry of neutralisation, 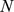
 **.** The concentration of spikes and antibodies is the same for all graphs, i.e. 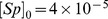 and 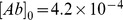 and the stoichiometry of entry is 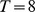. (A) All binding constants have the same value 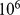 and all dissociation have the same value 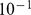. (B) Same coloured graphs correspond to the same reaction constants. Blue curves: the 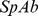-complex is built preferentially, due to the reaction constants 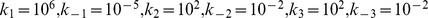. Red curves: the 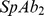-complex is built preferentially, 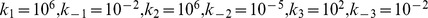. Green curves: the 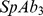-complexes are built preferentially, 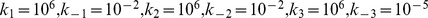. (C) The binding constants decrease and the dissociation constants increase, i.e. 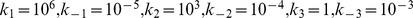. Only in this case are the kinetic neutralisation curves for different stoichiometries of neutralisation distinguishable.

**Figure 5:**
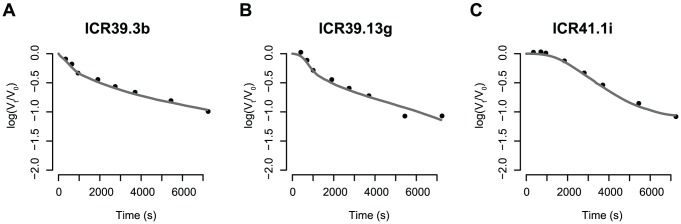
Simultaneous fit of the reaction constants and the stoichiometric parameters. Each panel shows the kinetic neutralisation curve (as predicted by equation 6) that best fitted kinetic neutralisation data. This data was extracted from [5] where three monoclonal rat antibodies against HIV-1 IIIB were tested: (A) ICR39.3b (B) ICR39.13g (C) ICR41.1i. The estimated parameters for each best fit are summarised in table 2.

The mathematical description of the reaction mechanism in equation 1 is so flexible that it allows for a wide range of reaction constants as well as reaction orders. To date neither the reaction constants nor the reaction orders are known for antibody binding reactions. Therefore, we first tested two canonical scenarios for the reaction orders with a wide range of reaction constants to find out which reaction orders lead to realistic predictions for kinetic neutralisation curves. (I) In the first scenario all reaction orders are one, i.e. 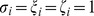 for 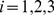. The chemical interpretation of this scenario is that all single step reactions are elementary reactions, we therefore refer to this model as *the elementary reaction model*. (II) The second scenario tested here reflects the (chemical) stoichiometic parameters of the overall reaction (equation 1), i.e. 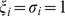 and 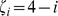 for 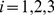. We therefore refer to this model as *the stoichiometric reaction model*. In the first scenario, the kinetic neutralisation curves drop very fast and either stays on a constant level or increases again. In Figure 2 we show three typical curves for this model. This behaviour is in contrast to the experimentally observed kinetic neutralisation curves. However, kinetic neutralisation curves of the second scenario can capture the real behaviour (see Figures 3–5 in which the stoichiometric reaction model is used and Figure 5 for data of kinetic neutralisation curves of monoclonal antibodies extracted from [5]). Therefore, we only consider these reaction orders in what follows.

#### Starting concentrations

The starting concentration of antibodies, 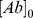 in comparison to the concentration of spikes determines the speed of the binding reaction and therefore the speed of neutralisation. The higher 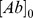 is, the faster is the neutralisation of viruses. In addition to the speed of neutralisation, the starting concentration of antibodies determines the final level of neutralisation. There is a certain threshold for the starting concentration below which neutralisation cannot reach 100%. This is the case when there are fewer antibodies than the average number of antibodies needed for neutralising all virions, which can be determined according to [22]. But even if the starting concentration of antibodies is bigger than this threshold, neutralisation does not necessarily reach 100% due to the equilibrium between antibody binding and dissociation. The higher the dissociation constant is, the faster antibodies fall off and the lower is the final level of neutralisation. Figure 3 (A) illustrates these findings which are robust for all stoichiometries of entry and stoichiometries of neutralisation as well as for all tested combinations of reaction constants.

#### Stoichiometry of entry

The stoichiometry of entry, 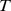, is the minimal number of viral spikes that engage with cellular receptors to mediate cell entry. A virion with 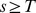 spikes is infectious if there are at least 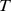 functional spikes. A trimer is functional if fewer than 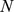 antibodies are bound. Contrariwise, a virion is neutralised if fewer than 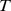 spikes remain functional, or in other words if at least 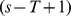 spikes have lost their functionality. Thus, the higher the stoichiometry of entry, 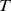, is, the fewer spikes must be neutralised for virion neutralisation. This is also reflected in the kinetic neutralisation curves in Figure 3 (B). The higher the stoichiometry of entry is, the faster the virions are neutralised and the higher is the final level of neutralisation (which is the same as lower levels of 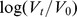). This finding holds true for all combinations of binding constants tested, and is consistent for all stoichiometries of neutralisation and starting concentrations of antibodies.

#### Reaction constants

Different monoclonal antibodies bind with different strength to the corresponding epitopes, i.e. binding and dissociation constants may vary between the different monoclonal antibodies. Early measurements of binding and dissociation rates for a mouse monoclonal antibody show that the binding rates range between 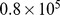 and 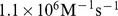 and the dissociation rates range between 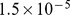 and 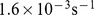 [28]. This study only determines the reaction constants for one antibody binding to a specific epitope. However, each HIV spike has three identical envelope proteins and therefore three antibody-specific epitopes. Due to steric hindrance or other effects, the reaction constants for the second and third antibody binding to a spike may differ from the reaction constants for the first antibody. We therefore test the influence of the reaction constants on the predictions for the kinetic neutralisation curves. In this analysis we focus on two aspects: (i) The impact of the ratio between binding and dissociation constants in the case when binding and dissociation constants each have the same value, i.e. 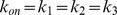, and 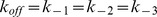. (ii) The impact of the reaction constants on the feasibility of parameter estimation.

#### What is the impact of the reaction constant ratio?

To answer this question, we calculated the kinetic neutralisation curves assuming that all binding constants are 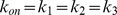 and all dissociation constants are 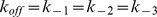. Figure 3 (C) shows five kinetic neutralisation curves for constant 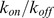 ratio. In this specific case (one binding site per antibody as well as constant binding and dissociation constants), the 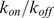 ratio equals the total overall reaction constant which is often referred to as affinity (the difference between affinity and avidity and its consequences are discussed in the Discussion section). The speed of neutralisation is the higher, the bigger the binding constants are. The same ratio of binding to dissociation constant does not lead to the same kinetic neutralisation curve but to the same final level of neutralisation. Figure 3 (D) shows the impact of the 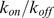 ratio. In this figure, the binding ratio is kept constant and the dissociation rates increase. The lower the dissociation rate and thus the bigger the ratio 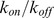 is, the faster viruses are neutralised. If the dissociation rate exceeds a certain threshold, antibodies fall off too quickly for guaranteed neutralisation. In summary, we see that the bigger the ratio between the sum of the binding parameters and the sum of the dissociation parameters is, the faster virus neutralisation happens.

#### Under which conditions is parameter estimation feasible?

To date, the reaction constants have not been identified. However, with our model framework it is possible to derive general rules for when the estimation of stoichiometric parameters is possible. In Figure 3 (B) we showed that the variation between kinetic neutralisation curves for different stoichiometries of entry, 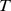, are quite big and therefore the estimation of this parameter may be possible. However, the reaction constants influence the ability to distinguish kinetic neutralisation curves for different stoichiometries of neutralisation, 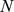. If the binding and dissociation constants are all equal for the three binding steps, most of the spikes will be bound to three antibodies and only a few to one or two antibodies. In this case, the kinetic neutralisation curves for different stoichiometries of neutralisation are the same (see Figure 4 (A)). If the binding and dissociation constants are such that formation of one spike-antibody complex (e.g. 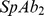) is fast and the formation of the others is slow, this spike-antibody complex forms an attraction point for the overall reaction. Most of the spikes are bound to that specific number of antibodies (e.g. if 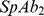 forms the attraction point, almost all spikes are bound to two antibodies). Figure 4 (B) shows kinetic neutralisation curves for different attraction points. If the attraction point is 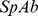, a virion population cannot be neutralised with antibodies having a 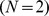
- or 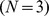-stoichiometry (dashed and dotted blue lines). If the attraction point is 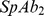, antibodies with a 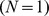
- and a 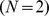-stoichiometry have the same kinetic neutralisation curve (solid and dashed red line). An antibody with a 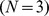
- stoichiometry, however, cannot fully neutralise the virion population but the neutralisation levels off (dotted red line). If the attraction point is 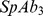, the kinetic neutralisation curves for all stoichiometries are the same (solid, dashed and dotted green line). If the binding constants decrease and the association constants increase (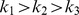 and 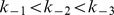), there is a time delay in the formation of spike antibody complexes with two and three antibodies respectively. This leads to different dynamics of the kinetic neutralisation curves for different stoichiometries of neutralisation. Thus, the kinetic neutralisation curves for different stoichiometries of neutralisation are clearly distinguishable and the estimation of this stoichiometric parameter might be possible.

### Comparing our predictions with earlier data

McLain and Dimmock [5] studied the kinetics of three monoclonal rat antibodies against HIV-1 IIIB. We extracted the kinetic neutralisation data to which we fitted our model of the percent infectivity (equation 6 where the fraction of spikes bound to 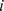 antibodies at time 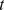, 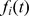, is calculated according to equations 2 and 3). To this end, we allowed the stoichiometry of entry to be 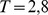 or 19 as found for different model assumptions in [20]. We further assumed the distribution of trimer numbers to follow the discretised B-distribution defined previously [20]. McLain and Dimmock [5] did not measure the virus concentration directly. The antibody concentrations are only shown in 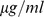. As we do not know the exact molar weight of these antibodies we cannot calculate the antibody concentration. In addition, it is not possible to reconstruct the exact virus (and hence the exact spike) concentration out of the syncytium-formation assay. Therefore, we used the starting concentrations 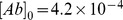 and 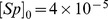 as above. The binding and dissociation rates are also not known. Therefore, we performed a non-linear regression on the data by simultaneously estimating the binding and dissociation constants and the stoichiometry of entry and neutralisation. Finally, we applied this fitting routine to the elementary reaction model and the stoichiometric reaction model.

The stoichiometric reaction model fits McLain and Dimmock's data [5] significantly better than the elementary reaction model. This is in accordance with the finding that the elementary reaction model does not reflect experimentally observed kinetic neutralisation curves and supports the conclusion that antibody binding reactions are not elementary reactions.

Figure 5 shows the best fits for the three different antibodies employing the stoichiometric reaction model. The stoichiometry of entry, 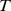, equals 2 for all three antibodies. The stoichiometry of trimer neutralisation is 1 for ICR39.3b and ICR39.13g and 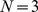 for ICR41.1i. The reaction constants are shown in Table 2. In parameter estimations as performed here, confidence intervals would be determined by a bootstrap routine. As there are only eight data points per antibody and eight variables to estimate, this method cannot be used to derive confidence intervals for the parameters. In addition, the statistical power of these estimates is not strong. However, our estimation procedure shows that the model framework can be applied to kinetic neutralisation data and can be used to estimate stoichiometric parameters. Applying the theoretical framework presented in [22], the average number of antibodies having to bind to one average virion is then 23 for ICR39.3b and ICR39.13g and 41 for ICR41.1i.

**Table 2:** Estimated parameters.

mAb	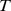	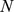	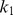	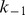	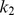	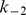	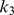	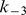	mean number of nAbs
ICR39.3b	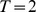	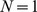	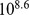	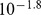	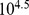	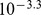	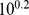	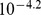	23
ICR39.13g	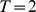	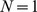	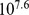	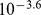	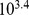	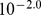	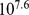	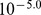	23
ICR41.1i	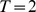	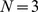	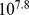	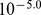	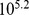	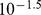	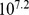	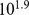	41

Estimates for the stoichiometry of entry, 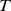, stoichiometry of neutralisation 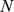 and the reaction parameters obtained by fitting the kinetic neutralisation curve model to experimental data for three rat monoclonal antibodies [5]. The kinetic neutralisation curves as well as the original data is shown in Figure 5. The mean number of antibodies needed for neutralisation is calculated as described in [22] and accounts for unproductive antibody binding. All dissociation constants have the unit 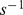 and the binding constant 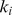 have the unit 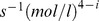, for 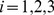.

### Other viral systems

Other viruses may have other strategies including different receptors or pathways like endocytosis (for an overview over different entry mechanisms see e.g. [29]). Even though the mechanisms for entry differ substantially, all viruses have to attach to cellular receptors via viral spikes as a first step in infection. These spikes are excellent targets for neutralising antibodies. For example, influenza type A virus is estimated to express 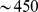 spikes on its surface [30]. Hemagglutinin, the spike responsible for viral entry [31], is also a trimeric protein, i.e. 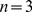 [32] but not all of the 450 spikes expressed on the virion's surface are hemagglutinin proteins. Hepatitis C Virus is a small (diameter 40–60 nm), enveloped virus. The viral spike that plays a major role in viral entry consists of two envelope proteins, E1 and E2, forming heterodimers [33], [34].

**Figure 6:**
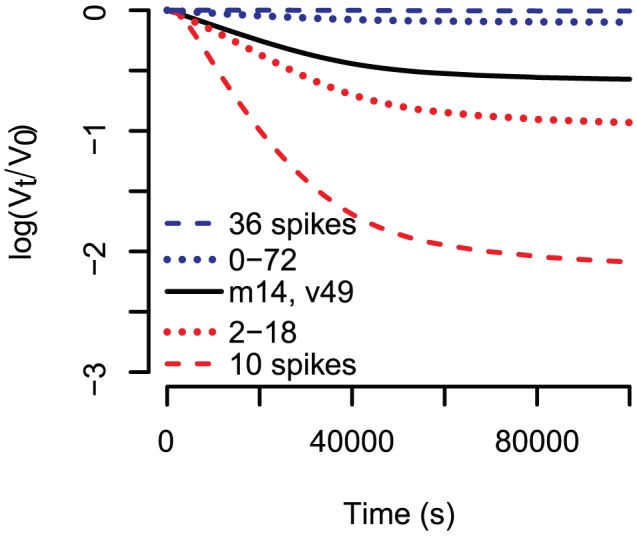
Kinetic neutralisation curves for different spike number distributions. Binding constants are all 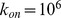, dissociation constants are all 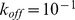, the stoichiometry of entry is 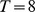 and the stoichiometry of trimer neutralisation is 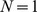. Red curves have a spike number distribution with mean 10, where all virions in the case of the dashed line have exactly 10 spikes and in case of the dotted lines have an equal probability to have 2,3…, 18 spikes. The black curve underlies the HIV specific discretised Beta distribution with mean 14 and variance 49. The spike number distributions for the blue curves have mean 36, where the one for the dashed line has only virions expressing 36 spikes and the dotted line has 0–72 spikes.

Our model (equations 2 and 6) is formulated with enough flexibility that we can account for variation in trimer number distribution and variation in binding sites within a trimer. However, we only test the effect of variation in the trimer number distribution here. In Figure 6 we show the kinetic neutralisation curves for different viral populations. Curves in red are based on virions with a mean trimer number distribution of 10, black 14 and blue 36. The higher the trimer number is, the slower neutralisation happens. This means the more spikes a virion expresses, the more antibodies must bind for neutralisation. The dashed red line and the dashed blue line are based on virions with exactly 10 and 36 spikes, respectively. The dotted red line is based on spike numbers varying from 2 to 18 and the dotted blue line 0–72 spikes. Comparing the dotted and the dashed lines, one sees that variation in spike numbers has an effect on the kinetic neutralisation curves. However, more variation in spike numbers does not necessarily means slower neutralisation.

## Discussion

In this paper we derive a model for antibody neutralisation that combines binding kinetics with stoichiometries. Antibodies bind to the viral surface spikes according to a simple chemical multi-step reaction. Whether a virion is still infective is defined via the concept of stoichiometries: at least 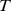 spikes must be bound to fewer than 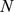 antibodies each. With this framework it is possible to predict published observations of kinetic neutralisation curves.

In the past, the interpretation of kinetic neutralisation curves was based on a theoretical framework derived in [2]. A straight decline in the time-log(percent infectivity) curve was interpreted as a single-hit mechanism, i.e. one antibody is sufficient to neutralise a whole virion. The main assumption of this theory was that there are critical and non-critical binding sites on the virion. As soon as an antibody binds to one critical site, the whole virion was assumed to be neutralised. However, in our model there is no need to subdivide the binding sites into critical and non-critical. The curvature is determined by the binding and dissociation constants. The former concept interpreted the levelling out as a persistent virus fraction. In our model, levelling out is due to antibody binding and dissociation kinetics.

The model for the percent infectivity (equation 6) depends on many parameters but we are mostly interested to estimate the stoichiometry of entry and neutralisation. Unfortunately, all other parameters can have a big influence on the predictions of the kinetic neutralisation curves. The reaction orders of antibody binding in the several binding steps are not known. We tested two simple scenarios here. As the binding of an antibody to an epitope involves huge binding sites and not only single atoms, the reactions might not follow simple elementary reaction principles and should be studied in more detail to confirm or neglect our assumed scenario. Besides the reaction orders, the most influential parameters are the binding and dissociation constants. They not only shape the curve at the beginning of the reaction but they also have a huge impact on whether it is possible to estimate the stoichiometry of trimer neutralisation from kinetic neutralisation curves. Therefore, we recommend to study these parameters in a different experimental setup. The starting concentration of antibodies and spikes can easily be measured at the beginning of the experiment. The spike concentration is equal to the number of virions times the average number of spikes per virion divided by the volume of the solution tested. The number of virions can be measured by quantitative real time polymerase chain reaction and the average number of spikes per virion by counting spikes on cryo-electron microscopical pictures of virions [26]. The estimation of the reaction constants and the stoichiometry of entry and neutralisation must be seen more as a proof of our method than a reliable estimate of stoichiometric parameters. An additional confounding factor might be non-functional spikes that are still able to bind antibodies. This would result in lower net antibody concentrations. If there are many non-functional spikes, this effect might have a non-negligible influence on the prediction of the percent infectivity with our model. We therefore recommend to quantify the amount of non-functional spikes. As soon as more data becomes available, our framework can be used to estimate stoichiometrical parameters.

In our study, we reanalysed kinetic neutralisation curves for three rat monoclonal antibodies against HIV-1 IIIB [5]. The virions of the human immunodeficiency virus express low trimer numbers (mean 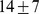 [7]) and the spikes responsible for entry consist of three identical gp120/gp41 subunits. We therefore assume that each monoclonal antibody binds with only one Fab-region and up to three antibodies can bind to one spike. Under these assumptions we also checked the influence of the trimer number distribution on the kinetic neutralisation curves. The higher the mean trimer number is, the slower virions are neutralised. Other viruses can differ in their route of entry, but entry always involves attachment to cellular receptors [29]. Antibodies binding to viral spikes can therefore at least theoretically confer neutralisation. This means that our framework can also explain neutralisation of other viruses. However, if the spike density exceeds a certain threshold, antibodies can bind with their second Fab region and the concept of avidity comes into play. The binding strength that exists between one Fab region and one epitope is called affinity. If the second Fab region of an IgG antibody binds to another epitope the binding strength between the antibody and the pathogen increases more than the twofold binding strength between one Fab region and one epitope. This enhanced binding strength is called avidity. For simplicity, we did not account for avidity in the model presented here. We explained why this can be done in the case of HIV earlier. For other viruses with a higher spike density, however, avidity may play an important role. In this case, our model must include more complex structures of antibody spike complexes in the form of 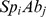. The number of binding and dissociation constants in this case will be increasing tremendously.

In our model, antibodies can bind and fall off any epitope. However, there are some HIV-antibodies that lead to irreversible destruction of the trimer [35]. Studying these antibodies with our model framework requires setting the dissociation rate to 0. If the destruction of the trimers happens when fewer antibodies bind to a spike than binding sites on the spike all dissociation rates of spike-antibody complexes with more than this threshold number must be set to 0.

In this study we focus on the analysis of kinetic neutralisation curves. Virologists normally characterise antibodies according to the concentration at which 50% of the neutralising effect is reached, the IC50. To this end, the neutralisation potential of antibody solutions of different concentrations are tested. We have shown, that the prediction of the kinetic neutralisation curves depend on the startimg concentration of antibodies. When defining a time point at which the percent infectivity should be measured, we can also adapt our model to predict titration curves. How well these predictions can be used for estimating stoichiometrical parameters is the subject of future studies.

So far, our results focus on *in vitro* systems with monoclonal antibodies. *In vivo* systems are far more complicated. The immune system elicits a huge variety of different antibodies with different reaction constants and different concentrations. In the future it will be necessary to study how different antibodies interact with each other, e.g. do they synergise or antagonise? It may also be possible that the binding of one antibody leads to conformational changes within the trimer leading to revelation of another epitope that is targeted by a more potent antibody. These mixtures of antibodies will require more elaborate models than the framework presented here. Similar to the concept of 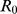 in epidemiology it might not be necessary to neutralise every single virion but reduce the amount of non-neutralised virions such that on average, each virion produces less than one offspring [36]. This might be already reachable with antibody concentrations that do not confer 100%neutralisation. However, whether a vaccine-induced antibody response or passive immunisation with antibodies lead to full neutralisation of all virus particles *in vivo* also depends on the concentration of virions and antibodies across different body compartments, such as blood or mucosal surfaces. The virion concentration as well as the antibody concentration could vary substantially from compartment to compartment and the antibody concentration might not be sufficient for neutralisation in some of them.

To date, the presented framework still needs conformation by experimentalists. As pointed out above, the most important experiment to be done is the determination of the reaction constants. Once these are available, our framework can be used to infer stoichiometries. With the help of stoichiometries it is possible to determine the numbers of antibodies needed for neutralisation *in vitro*. If the antibodies behave similarly *in vivo*, our models make it possible to compare different antibodies on a rational basis as soon as the stoichiometrical values will have been determined for different antibodies. By extending our framework, it might be possible to also study interactions between different antibodies more rationally which will complete the picture of antibody based neutralisation.

## Supporting Information

Dataset S1 Source code for the calculations described in the main text. The file can be sourced into R with the command ‘source(“<direction of file>/sourcecode.R”)’ and the commented examples can then be executed.
(R) Click here for additional data file.
